# Neurocognitive correlates of semantic memory navigation in Parkinson’s disease

**DOI:** 10.1038/s41531-024-00630-4

**Published:** 2024-01-09

**Authors:** Felipe Diego Toro-Hernández, Joaquín Migeot, Nicolás Marchant, Daniela Olivares, Franco Ferrante, Raúl González-Gómez, Cecilia González Campo, Sol Fittipaldi, Gonzalo M. Rojas-Costa, Sebastian Moguilner, Andrea Slachevsky, Pedro Chaná Cuevas, Agustín Ibáñez, Sergio Chaigneau, Adolfo M. García

**Affiliations:** 1 Graduate Program in Neuroscience and Cognition, Federal University of ABC, São Paulo, Brazil; 2 Center for Social and Cognitive Neuroscience, School of Psychology, Universidad Adolfo Ibáñez, Santiago, Chile; 3 Latin American Brain Health Institute, Universidad Adolfo Ibáñez, Santiago, Chile; 4 Laboratorio de Neuropsicología y Neurociencias Clínicas, Universidad de Chile, Santiago, Chile; 5 Cognitive Neuroscience Center, Universidad de San Andrés, Buenos Aires, Argentina; 6 National Scientific and Technical Research Council, Buenos Aires, Argentina; 7 Facultad de Ingeniería, Universidad de Buenos Aires, Buenos Aires, Argentina; 8 Global Brain Health Institute, University of California, San Francisco, California, USA; & Trinity College, Dublin, Ireland; 9 Department of Radiology, Clínica las Condes, Santiago, Chile; 10 Advanced Epilepsy Center, Clínica las Condes, Santiago, Chile; 11 Join Unit FISABIO-CIPF, Valencia, Spain; 12 School of Medicine, Finis Terrae University, Santiago, Chile; 13 Health Innovation Center, Clínica Las Condes, Santiago, Chile; 14 Memory and Neuropsychiatric Center (CMYN), Neurology Department, Hospital del Salvador & Faculty of Medicine, University of Chile, Santiago, Chile; 15 Geroscience Center for Brain Health and Metabolism (GERO), Santiago, Chile; 16 Neuropsychology and Clinical Neuroscience Laboratory (LANNEC), Physiopatology Program – Institute of Biomedical Sciences (ICBM), Neuroscience and East Neuroscience Departments, Faculty of Medicine, University of Chile, Santiago, Chile; 17 Neurology and Psychiatry Department, Clínica Alemana-Universidad Desarrollo, Santiago, Chile; 18 Facultad de Ciencias Médicas, Universidad de Santiago de Chile, Santiago, Chile; 19 Center for Cognition Research, School of Psychology, Universidad Adolfo Ibáñez, Santiago, Chile; 20 Departamento de Lingüística y Literatura, Facultad de Humanidades, Universidad de Santiago de Chile, Santiago, Chile

**Keywords:** Diagnostic markers, Parkinson's disease

## Abstract

Cognitive studies on Parkinson’s disease (PD) reveal abnormal semantic processing. Most research, however, fails to indicate which conceptual properties are most affected and capture patients’ neurocognitive profiles. Here, we asked persons with PD, healthy controls, and individuals with behavioral variant frontotemporal dementia (bvFTD, as a disease control group) to read concepts (e.g., ‘sun’) and list their features (e.g., *hot*). Responses were analyzed in terms of ten word properties (including concreteness, imageability, and semantic variability), used for group-level comparisons, subject-level classification, and brain-behavior correlations. PD (but not bvFTD) patients produced more concrete and imageable words than controls, both patterns being associated with overall cognitive status. PD and bvFTD patients showed reduced semantic variability, an anomaly which predicted semantic inhibition outcomes. Word-property patterns robustly classified PD (but not bvFTD) patients and correlated with disease-specific hypoconnectivity along the sensorimotor and salience networks. Fine-grained semantic assessments, then, can reveal distinct neurocognitive signatures of PD.

## Introduction

Parkinson’s disease (PD) is the most prevalent and fastest growing movement disorder worldwide^1^. Cognitive research on the condition underscores semantic assessments as a scalable approach to identify sensitive markers^2–4^. Yet, most studies only compare response accuracy or speed relative to healthy controls (HCs)^5^, failing to reveal which semantic features typify patients’ conceptual structures and capture their neurocognitive profiles. Moreover, few studies include disease control groups. Valuable insights are thus missing for clinical characterization and neuropsychological modeling of the disorder. To address these challenges, we recruited PD patients, HCs, and persons with behavioral variant frontotemporal dementia (bvFTD, another condition involving non-primary semantic deficits);^6,7^ analyzed lexico-semantic features of their responses to a property listing task; and examined whether such features correlated with their cognitive and neurofunctional profiles.

Core motor symptoms in early stages of PD are typically accompanied by cognitive dysfunctions^8^, including semantic anomalies^3,9^. These range from difficulties with concept association^10^, retrieval^11^, and comprehension^4,10,12,13^ to word finding and definition deficits^14^. Such impairments can emerge preclinically;^2,15^ discriminate between patients with different cognitive profiles^3^ and medication status;^16^ and correlate with abnormalities in frontostriatal^17,18^, perisylvian^19^, prefrontal, and anterior cingulate^20,21^ hubs along the sensorimotor, semantic, and salience networks. Thus, semantic assessments could support mainstream clinical testing in this population.

Yet, most studies measure performance by counting or timing correct responses, overlooking how patients construe concepts as they navigate semantic memory. This is a critical gap, as meta-analytical evidence shows that word production deficits in PD cannot be reduced to dysarthria or low processing speed^22^. Promisingly, analyses of responses’ word-level properties reveal distinct disturbances in PD and other neurodegenerative disorders^23–25^. Specifically, two semantic features may be particularly sensitive to PD: concept abstraction and semantic variability.

First, a concept’s abstraction depends on the concreteness (sensory characteristics) and imageability (ease of mental visualization) of its real-world referents^26^. Highly concrete and imageable concepts (e.g., ‘table’, as opposed to ‘freedom’) involve faster responses^27^ as well as reduced electrophysiological^28^ and hemodynamic^29^ brain modulations, pointing to lower cognitive effort. In PD research, concreteness and imageability values capture subtle differences between patients and HCs^9^. Interestingly, abstract concept processing is affected in cognitively impaired PD patients but often spared in cognitively preserved ones^3,11,12^, revealing a link with overall cognitive status. Also, difficulties with abstract words in PD are paralleled by decreased resting-state functional connectivity of the semantic^30,31^ and sensorimotor^32^ networks, highlighting their relevance for neurocognitive characterizations of the disease.

Second, semantic variability refers to changes in conceptual distance across successive words. This feature seems reduced in PD. Relative to HCs, patients exhibit semantically closer choices^23,33^, increased semantic priming^34^, and fewer semantically different clusters^35^. Since semantic variability requires suppressing the current semantic field to activate another, these patterns may reflect poor semantic inhibition, a typical trait of PD^36,37^. Indeed, semantic search skills and semantic distance between words are related to connectivity of the salience network^37,38^, which underpins cognitive inhibitory skills^39–41^ and whose impairments in PD predict reduced switching across concepts^42^.

Key correlates of these features can be further illuminated by comparisons with bvFTD, a disorder which also features non-primary semantic deficits^43^ and which partly shares linguistic, behavioral, and cognitive features with PD^6,7^. On the one hand, indirect evidence suggests that the proposed preference for less abstract concepts in PD may not be mirrored in this disease^4,44^. On the other, semantic variability may be similarly altered in bvFTD, which also involves marked inhibitory deficits^45^ and disruptions of key correlates, such as the salience network^46^. More generally, while lexico-semantic deficits are pervasive in PD^2,47^, they emerge less consistently in bvFTD^43,48^, likely supporting individual patient identification in the former but not in the latter population. Yet, semantic comparisons between these disorders remain incipient^4^, calling for novel evidence.

**Fig. 1:**
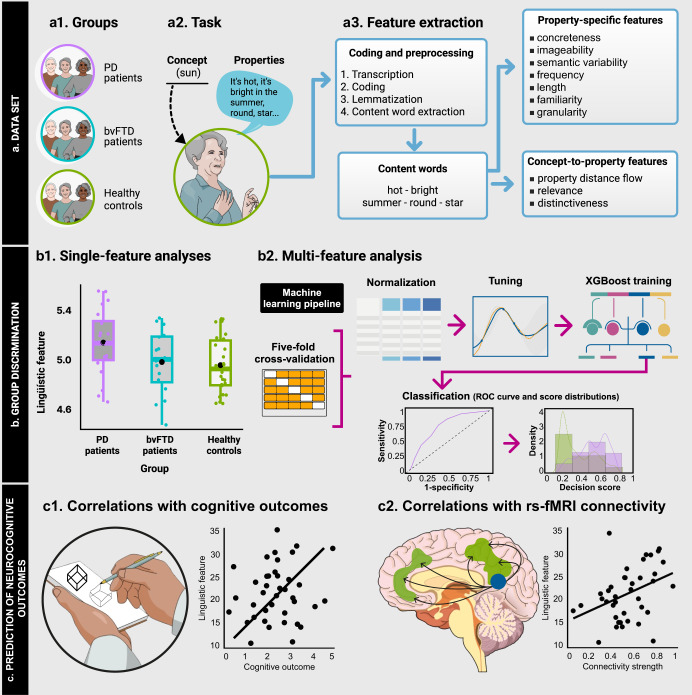
Task, pre-processing and analysis pipeline. **a** PD and bvFTD patients, as well as HCs (a1), performed a property listing task (a2) yielding diverse lexico-semantic features (a3). **b** Extracted features were compared between patients and HCs separately via ANOVAs (b1), and jointly via machine learning analysis (b2). **c** Discriminatory features were then correlated with cognitive outcomes (c1) and rs-fMRI connectivity (c2). This figure is an original creation from the authors. The images depicting human individuals (insets a1 and a2), human hands (inset c1), and a human brain (inset c2) were obtained through a paid subscription from Mind the Graph, in compliance with a CC BY-SA 4.0 DEED Attribution-ShareAlike 4.0 International license allowing for unrestricted use of the material (https://creativecommons.org/licenses/by-sa/4.0/).

Here we examined semantic markers in 20 PD and 16 bvFTD patients, compared with 26 HCs, through a validated property listing task requiring free description of concepts^49,50^. Responses were analyzed in terms of concreteness, imageability, semantic variability, and other lexico-semantic properties reported in neurodegeneration research^33,51–53^. These features were compared between groups separately (via inferential statistics) and jointly (via machine learning analysis). The latter approach employed a stratified five-fold cross-validation using XGBoost^54^, after min-max normalization and hyperparameter tuning^55^. The most discriminative features were then correlated with cognitive status and semantic inhibition measures, as well as with brain connectivity patterns along the sensorimotor, semantic, and salience networks. Our research design is diagrammed in Fig. 1.

We raised four sets of hypotheses. First, we predicted that only PD patients would produce more concrete and imageable words than HCs, and that both patient groups would exhibit reduced semantic variability. Second, we hypothesized that machine learning analysis of semantic features would robustly identify PD patients, but not bvFTD patients. Third, we anticipated that concreteness and imageability would correlate with patients’ general cognitive status as well as disruptions along the sensorimotor and/or the semantic networks. Finally, we expected semantic variability to correlate with semantic inhibition deficits and salience network connectivity. By testing these hypotheses, we aim to illuminate the neurocognitive particularities of semantic processing in PD.

## Results

### Single-feature analyses

**Fig. 2:**
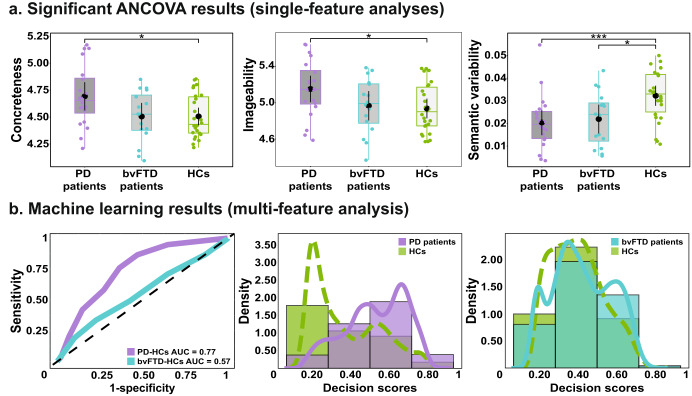
Statistical analyses and results. **a** Significant ANCOVA results showed differences between PD patients and HCs in only three features (concreteness, imageability, and semantic variability). Differences between bvFTD patients and HCs were found in semantic variability. No differences were found between patient groups. Boxplots display the mean, median, interquartile range, and range of each variable. **b** Subject-level classification was robust between PD patients (purple) and HCs (sky blue) (AUC = 0.77) but not between bvFTD (green) and HCs (AUC = 0.56).

ANCOVA results (Fig. 2a) revealed main effects of concreteness (*F*_2,58_ = 4.27, *p* = 0.019, η_p_^2^ = 0.08), imageability (*F*_2,58_ = 3.42, *p* = 0.039, η_p_^2^ = 0.06), and semantic variability (*F*_2,58_ = 6.98, *p* < .01, η_p_^2^ = 1.90). Post hoc comparisons, via Tukey’s HSD tests, showed significant differences between PD patients and HCs in all three variables (concreteness: *p* = 0.027, *d* = 0.78; imageability; *p* = 0.039, *d* = 0.74; semantic variability: *p* < 0.01, *d* = 1.04). Differences between bvFTD patients and HCs were significant for semantic variability (*p* = 0.020, *d* = 0.91), but not for concreteness (*p* < 0.99, *d* < 0.01) or imageability (*p* = 0.943, *d* = 0.11). Contrasts between PD and bvFTD patients did not reach significance in any of these variables (all *p*-values > 0.054). No other variable yielded significant main effects of group (all *p*-values > 0.054). For details, see Supplementary material 1.

### Multi-feature analysis

Considering all property-specific and concept-to-property features together, classification was successful between PD patients and HCs (AUC = 0.77) but not between bvFTD patients and HCs (AUC = 0.56). AUC scores are shown in Fig. 2b (left inset) and associated decision scores are shown in Fig. 2b (middle and right insets). For details, see Supplementary material 2.

### Correlations with clinical measures

In the PD-HC tandem, concreteness (Spearman: *rho* = −0.34, *p* = 0.022) and imageability (Pearson: *r* = −0.30, *p* = 0.046) were negatively correlated with MoCA scores, while semantic variability was negatively correlated with Hayling scores (Spearman: *rho* = −0.30, *p* = 0.045). In the bvFTD-HC tandem, semantic variability was negatively correlated with Hayling scores (Spearman: *rho* = −0.32, *p* = 0.045). Every other correlation tested (including correlations with the total PDQ-39 score and the PDQ-39 mobility score) was non-significant (Supplementary material 3).

### FMRI connectivity differences and correlations with word-property measures

**Fig. 3:**
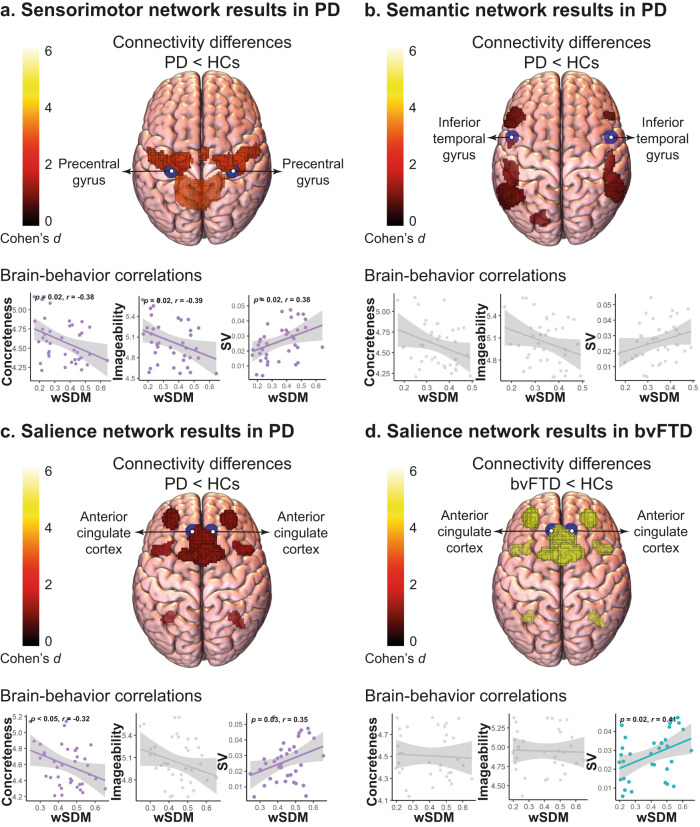
Resting-state fMRI connectivity results. PD patients presented hypoconnectivity in the (**a**) sensorimotor, (**b**) semantic, and (**c**) salience networks, whereas bvFTD patients exhibited selective hypoconnectivity in (**d**) salience network (networks’ colors indicate the effect size of the difference from HCs, expressed as Cohen’s *d*, ranging from 0 [black] to 6 [white]). In the PD-HC tandem, concreteness negatively correlated with connectivity of the sensorimotor and salience networks, imageability negatively correlated with sensorimotor network connectivity, and semantic variability positively correlated with salience network connectivity. In the bvFTD-HC tandem, semantic variability positively correlated with salience network connectivity. Non-significant correlations are shown with a gray mask. bvFTD: behavioral variant frontotemporal dementia; HCs: healthy controls; PD: Parkinson’s disease; SV: semantic variability; wSDM: weighted Symbolic Dependence Metric.

Compared to HCs, PD patients presented hypoconnectivity in the sensorimotor (*p* < 0.001, *d* = 4.05), salience (*p* < 0.001, *d* = 1.63), and semantic (*p* < 0.001, *d* = 1.45) networks. Persons with bvFTD also exhibited hypoconnectivity in the salience network (*p* < 0.001, *d* = 5.31). No other significant pairwise comparisons emerged for any other network (all *p*-values > 0.05). No network showed hyperconnectivity in any patient group (all *p*-values > 0.05) (Fig. 3).

In the PD-HC tandem, concreteness negatively correlated with connectivity of the sensorimotor (*p* = 0.02, *r* = −0.38) and salience (*p* < 0.05, *r* = −0.32) networks. Imageability negatively correlated with the connectivity of the sensorimotor network (*p* = 0.02, *r* = −0.39). Finally, semantic variability is positively correlated with the connectivity of the sensorimotor (*p* = 0.02, *r* = 0.38) and salience (*p* = 0.03, *r* = 0.35) networks. No other significant correlations emerged (Fig. 3, Supplementary material 4).

In the bvFTD-HC tandem, semantic variability positively correlated with the connectivity of the salience network (*p* = 0.02, *r* = 0.41). No other significant correlations emerged (Fig. 3, Supplementary material 3).

## Discussion

We aimed to identify neurocognitive markers of PD, vis-à-vis bvFTD, using word property analyses in a semantic task. While direct comparisons between PD and bvFTD did not reveal any significant differences, each group presented distinct profiles when compared with HCs. Specifically, PD patients exhibited anomalies in concreteness, imageability, and semantic variability, whereas bvFTD patients presented alterations only in semantic variability. Joint machine learning analyses of these and other word properties discriminated PD (but not bvFTD) patients from HCs. Concreteness and imageability correlated with cognitive status in PD, whereas semantic variability values correlated with inhibition outcomes in both disorders. Alterations of these three variables were associated with hypoconnectivity of the sensorimotor and the salience networks. These findings illustrate the relevance of semantic assessments to reveal neurocognitive signatures of PD.

PD patients’ responses were characterized by higher concreteness and imageability. This mirrors results from statistical learning analyses showing that both variables contribute to discriminating PD patients from HCs^9^. Given that increased concreteness and imageability involve reduced cognitive demands^27–29,56–59^, such findings suggest that patients favor easily accessible units during semantic memory navigation.

*Prima facie*, these results might seem to contradict well-established difficulties of PD patients in processing action concepts, typified by high concreteness and imageability^2^. However, action-concept deficits are predominant only in cognitively *unimpaired* cohorts, as abstract concept abnormalities are pervasive in cognitively *impaired* patients during productive^3^ and receptive^12^ language tasks. In this sense, our sample’s mean MoCA score fell slightly below the cutoff for mild cognitive impairment, within a range similar to that reported in previous PD research from Chile and other underrepresented regions for patients of similar ages^23,60–64^. This supports the relevance of abstract concept anomalies for this disease phenotype^3,12^. Moreover, concreteness and imageability values correlated negatively with MoCA scores, indicating that the greater the cognitive impairment, the greater the reliance on highly accessible sub-domains within semantic memory. Therefore, fine-grained conceptual analysis seems useful to tap into cognitive (dys)function in PD.

This group also exhibited reduced semantic variability –i.e., more consistent semantic distance across successive words. Earlier reports have shown that, relative to HCs, persons with PD produce semantically closer and less varied concepts^33^ as well as fewer semantic clusters^35^. Moreover, this population exhibits hyper-priming effects between sequential stimuli, suggesting abnormally high activation of preceding conceptual features^34,65^. In line with such findings, our results support the view that PD involves deficits in suppressing previous semantic information, arguably due to broad inhibitory disruptions^34^. Indeed, semantic variability in our study correlated with semantic inhibition skills (as captured by Hayling scores), extending evidence of associations between neurophysiological indices of semantic integration effort (viz., N400 modulations) and inhibitory control measures in PD^66^. Thus, current and previous findings suggest that difficulties with inhibiting active conceptual fields would favor less diverse semantic choices as patients navigate their vocabulary.

Machine learning analyses showed that semantic information was also robust for identifying *individual* PD patients from HCs. Upon combining concreteness, imageability, and semantic variability data with additional response properties, we discriminated between persons in each group with an AUC of 0.77. Robust classification of PD patients and HCs through semantic information has been previously reported via spontaneous speech^67^, picture description^16^, story retelling^3^, and verbal fluency^42^ tasks. Our study extends such findings by showing that PD patients may also be identified through a brief paradigm that directly taps on how patients construe concepts. Overall, these results underscore the utility of semantic analyses to reveal candidate cognitive markers of PD.

Brain-behavior associations reinforce this claim. First, concreteness, imageability, and semantic variability values in PD were associated with sensorimotor network connectivity, which was reduced across patients. Given that the sensorimotor network underpins lexico-semantic functions (e.g., word retrieval and reading)^68,69^ and is markedly compromised in PD^32^, such result suggests that word-property analysis may capture distinct neurocognitive anomalies in this population. Second, concreteness and semantic variability were associated with salience network connectivity, which was also decreased in our PD sample. Compatibly, the salience network has been implicated in semantic search abilities and semantic distance between successive words^37,38^, two key domains involved in the features at hand. Moreover, this network underpins cognitive inhibitory skills^39–41^ and its disruptions in PD correlate with diminished conceptual switches^42^, further supporting the hypothesis that reduced semantic variability is linked to poor inhibition skills. Finally, although the semantic network also exhibited hypoconnectivity in the PD group, it was not correlated with any word property. Tentatively, this might partly reflect the patients’ favoring of concrete over abstract semantic units, as the former would rely less on semantic network hubs, such as the anterior temporal lobe^69,70^ –although this link is not fully systematic^71,72^. In short, while further research is required, semantic assessments may also be sensitive to neurofunctional disruptions in PD.

Of note, persons with bvFTD did not exhibit alterations of concreteness or imageability relative to HCs, and these features did not correlate with their overall cognitive status. Moreover, machine learning analysis of all properties failed to discriminate between bvFTD patients and HCs (AUC = 0.56). Interestingly, however, the bvFTD group did mirror PD patients in showing lower semantic variability than HCs, corroborating that this variable is sensitive to disinhibition and salience network integrity. In fact, as in PD, such pattern correlated with both inhibitory disruptions and salience network hypoconnectivity, two salient features of bvFTD^45,73^. Even though direct comparisons between PD and bvFTD failed to yield significant differences, the observation of dissimilar and shared patterns in these groups relative to HCs enhances the cognitive profiling of both disorders^2,4,43^. In particular, while measures of concreteness and imageability may be particularly useful for PD screenings, semantic variability analysis might inform the clinical characterization of both PD and bvFTD.

The findings above bear clinical implications. Verbal semantics is often overlooked in routine PD assessments, echoing the classical view that language is unaffected in PD^74^. However, accruing evidence attests to the relevance of semantic measures for patient characterization, differential diagnosis, phenotyping, and monitoring^2–4,10–14,47,75,76^. Word-property analyses could fruitfully complement more expensive and invasive markers (such as those offered by biochemical, genetic, and neuroimaging tests)^77^ and more typical approaches to word-production assessments (e.g., valid response counts in verbal fluency tasks)^12^. In particular, the objectivity of normative semantic data (e.g., for concreteness and imageability) and natural language processing tools (e.g., to calculate semantic variability) could circumvent the biases and limitations of subjective examiner ratings^3,16^. More generally, these approaches could be a valuable addition to the recent call for more systematic screening of cognitive function in standard PD assessments^78^.

Of course, the present study involves labor-intensive data curation methods. Yet, its value does not lie on its current procedure but rather on the *discovery* of novel markers for ulterior clinical implementation. In this sense, open-source automated technologies are available for all our data processing steps, including recording and transcription^79^, part-of-speech tagging and lemmatization^80^, and feature extraction^81^. Moreover, generative artificial intelligence tools can now identify and remove task-irrelevant segments via prompt engineering^82^. Indeed, different speech and language analysis methods have already been incorporated into clinician-friendly apps for different disorders (including PD)^83–86^. Such antecedents illustrate the next steps to further develop our approach.

Importantly, our sample’s mean age and age at onset were roughly 74 and 72, respectively. While similar values have been reported^87^, earlier ages of onset (around 60) are common in the literature^88^. This raises the question of whether the reported markers would also prove robust in younger cohorts. In addition, given that our sample’s mean MoCA score fell below the population-specific cutoff for mild cognitive impairment, it would be vital to replicate our study with cognitively preserved patients, who actually represent approximately 70% of the population^89–91^. Both points open exciting avenues for further research.

We further note that our focus on Spanish-speaking Latinos meets the pressing need for research on underrepresented language groups^92^. Indeed, these individuals’ sociodemographic, cognitive, and daily living profiles may differ from those observed in high-income countries^93^. The effort is all the more worthwhile considering that well-established semantic memory assessments, such as the Cambridge Semantic Memory Test Battery, are available only in English^94^. Yet, we acknowledge that this precludes claims on our findings’ cross-linguistic generalizability. Promisingly, PD studies have shown semantic memory deficits in several other languages^14,30,31,33–35,37,38,42^ and relevant normative databases exist for many of them^95–100^. This scenario paves the way for informative replications of our approach across diverse speech communities.

Its contributions notwithstanding, our work features some limitations which pave the way for additional future work. First, our sample size was moderate. Although robust findings have been reported in relevant studies with similar numbers of participants^9,16,17,20,23,24,34,37,75,101,102^, replications with larger groups would be desirable. Second, our stimuli comprised concrete concepts only, precluding insights on other critical categories, such as action verbs^2,3^. Further studies should incorporate this and other sensitive concept types. Third, neuroimaging analyses were restricted to resting-state recordings. Online neuroimaging protocols would be needed to better understand the neural correlates of semantic processing in PD. Finally, key insights on the sensitivity and specificity of these candidate markers could be obtained through comparisons with Alzheimer’s disease, a neurodegenerative disorder involving pervasive and primary semantic memory deficits^23^.

In conclusion, this study introduced a new approach to examine semantic memory navigation in PD. Our findings suggest that word-property analysis in a brief conceptual task could contribute to patient characterization, discrimination, and neurocognitive monitoring. Further work along these lines could inform the global quest for scalable, equitable, and discriminatory markers of the disease.

## Methods

### Participants

The study comprised 62 right-handed^103^ native Spanish speakers from Chile: 20 PD patients, 16 bvFTD patients, and 26 HCs (Fig. 1a1). This sample size was adequate to obtain reliable effects, reaching a power of 0.98 (Supplementary material 5). PD patients were diagnosed following UKPD-SBB standards^104^ and tested in the ‘on’ phase of antiparkinsonian medication. None of them had Parkinson-plus symptoms nor a history of deep brain stimulation. Patients with bvFTD were diagnosed following current criteria^45^. They all exhibited sociobehavioral deficits, as defined by caregivers^105–107^, and presented with atrophy in canonical frontal regions. Both patient groups and HCs had normal or corrected-to-normal hearing and vision. Diagnoses were supported by an extensive neurological, neuropsychiatric, and neuropsychological examination, as in previous works^4,108^. No patient reported a history of other neurological disorders, psychiatric conditions, primary language deficits, or substance abuse. HCs were cognitively preserved as well as functionally autonomous, and they reported no history of neuropsychiatric disease or alcohol/drug abuse.

The three groups were matched for sex, age, and occupation, but not for education, so this variable was entered as a covariate in all behavioral data analyses. PD and bvFTD patients were also matched for time since diagnosis. All participants were further characterized in terms of overall cognitive status through a Chilean validation Montreal Cognitive Assessment (MoCA)^109^. This instrument revealed cognitive compromise in the PD group, given the cutoff of 21 points for mild cognitive impairment^60^. General health status was assessed via the Parkinson’s Disease Questionnaire (PDQ-39), which taps on eight dimensions of daily living^110^. Motor functionality was evaluated with the PDQ-39 mobility subscale^110^. PDQ-39 scores revealed low general health status and motor functionality in PD patients. Cognitive inhibition skills were evaluated with a validated version of the Hayling test^111^. Demographic and neuropsychological data are detailed in Table 1. All participants signed an informed consent according to the Declaration of Helsinki, and the study was approved by the local Ethics Committee.

**Table 1:** Demographic and neuropsychological information.

	PD *N* = 20	bvFTD *N* = 16	HCs *N* = 26	Main Effect	Pairwise comparisons (for significant main effects)	
					Groups	*p*-value
**Demographic data**						
Sex (F:M)	10:10	5:11	16:10	χ^2^ = 3.64 *p* = 0.162^b^	HCs-PD HCs-bvFTD PD-bvFTD	----- ----- -----
Age	74.25 (6.46) [62–89]	68.50 (12.27) [42–87]	71.73 (5.09) [63–80]	*F* = 2.35 *p* = 0.105^a^	HCs-PD HCs-bvFTD PD-bvFTD	----- ----- -----
Occupation (R:A)	13:7	9:6	13:11	χ^2^ = 0.534 *p* = 0.766^b^	HCs-PD HCs-bvFTD PD-bvFTD	----- ----- -----
Years since diagnosis	2 (2.21) [0–10]	2.79 (2.59) [0.25-8]	-----	*t* = -0.890 *p* = 0.381^d^	-----	-----
Years of education	9.95 (5.10) [0–19]	14 (5.28) [6–21]	13 (3.77) [3–18]	*F* = 3.94 *p* = 0.025^b^	HCs-PD HCs-bvFTD PD-bvFTD	0.077^c^ 0.777^c^ 0.031^c^
**Neuropsychological and functional data**						
MoCA	20.05 (4.55) [11–27]	22.07 (4.80) [11–29]	24.33 (2.99) [18–29]	*F* = 6.33 *p* < 0.01^a^	HCs-PD HCs-bvFTD PD-bvFTD	< 0.01^c^ 0.214^c^ 0.331^c^
					HCs-PD HCs-bvFTD PD-bvFTD	0.030^c^ 0.027^c^ 0.956^c^
Hayling test	14.90 (10.30) [0-33]	15.79 (11.70) [4–37]	8 (5.18) [2–22]	*F* = 5.01 *p* < 0.01^a^		
PDQ-39 total score	72.19(24.68) [41–128]	-----	-----	-----	-----	-----
PDQ-39 mobility score	19.53 (9.72) [10–42]	-----	-----	-----	-----	-----

Data presented as mean (SD) [range], except for sex and occupation.

*PD* Parkinson’s disease, *bvFTD* behavioral variant frontotemporal dementia, *HCs* healthy controls, *MoCA* Montreal Cognitive Assessment, *PDQ-39* Parkinson’s Disease Questionnaire, *R* retired, *A* active.

^a^*p* value calculated via one-way ANOVA.

^b^*p* value calculated via chi-squared test (χ2).

^c^*p* value calculated via Tukey’s HSD test.

^d^*p* value calculated via two-tailed *t* test.

### Stimuli

Stimuli consisted in 10 Spanish words between one and three syllables (*M* = 2.1, *SD* = 0.57). The words denoted common natural entities and artifacts, namely, ‘tree’, sun’, ‘clown’, ‘puma’, ‘airplane’, ‘hair’, ‘duck’, ‘house’, ‘shark’, and ‘bed’ (in Spanish: ‘árbol’, ‘sol’, ‘payaso’, ‘puma’, ‘avión’, ‘pelo’, ‘pato’, ‘casa’, ‘tiburón’, ‘cama’). Normative data from EsPal (Duchon, et al., 2013) showed that these items ranked high in logarithmic frequency (*M* = 1.50, *SD* = 0.62), imageability (*M* = 6.44, *SD* = 0.36), concreteness (*M* = 6.13, *SD* = 0.32), and familiarity (*M* = 6.40, *SD* = 0.39). Such features rendered stimuli easily retrievable, as required to elicit rich responses in our target populations^112–114^.

### Procedure

The property listing task was conducted in a dimly illuminated soundproof room. Participants were asked to sit comfortably on a chair, close to a recording device. In line with reported procedures^115^, participants were asked to name as many *properties* of each concept as possible. They were told that these included physical characteristics, internal parts, appearance, sounds, smells, textures, uses, functions, and typical locations (Fig. 1a2). Each word was presented orally in fully randomized order. Examiner interventions were restricted to addressing clarification questions (e.g., “Can I say what the color of that is?”). No feedback was given after the answers. Participants were asked to confirm that they were done describing each stimulus before the following one was presented. The whole procedure lasted approximately 10 minutes.

### Response coding and preprocessing

Properties consisted in either single words or short phrases^50^. Each property was transcribed into a single cell in a spreadsheet. We excluded all non-valid responses, including statements directed to the examiner (e.g., for ‘hair’, ‘*yours is very pretty!*’), personal life experiences (e.g., ‘*I get a haircut very often*’), and metacognitive comments (e.g., ‘*What was the name of that cartoon with the duck?*’). Incorrect responses (e.g., for ‘puma’, ‘*has wings*’) and repetitions were deemed valid given their potential to illuminate aspects of the patients’ semantic processing. The mean number of valid responses per group is offered in Table 2. Transcriptions and coding were supervised and double-checked by a team of psychologists and linguists, all native Spanish speakers^116^. Every word in each valid property was then lemmatized with Python’s TreeTagger library (https://www.cis.lmu.de/~schmid/tools/TreeTagger/). Finally, lemmatized content words (nouns, verbs, adjectives, adverbs) were isolated for feature extraction. The mean number of content words per group is also listed in Table 2.

**Table 2:** Mean number of valid properties.

	PD *N* = 20	bvFTD *N* = 16	HCs *N* = 26	Main Effect	Pairwise comparisons	
					Groups	*p*-value
Valid properties	6.30 (2.35)	5.98 (2.82)	8.31 (2.96)	*F* = 5.49 *p* < 0.01^a^	HCs-PD HCs-bvFTD PD-bvFTD	0.027^b^ 0.015^b^ 0.926^b^
Content words	14.45 (7.74)	15.45 (9.35)	22.75 (10.77)	*F* = 5.93 *p* < 0.01^a^	HCs-PD HCs-bvFTD PD-bvFTD	<0.01^b^ 0.033^b^ 0.940^b^

Data presented as mean (*SD*), except for sex and occupation.

*PD* Parkinson’s disease, *bvFTD* behavioral variant frontotemporal dementia, *HCs* healthy controls.

^a^*p* value calculated via one-way ANCOVA.

^b^*p* value calculated via Tukey’s HSD test.

### Feature extraction

Participants’ responses were analyzed (Fig. 1a3) to extract property-specific features (capturing characteristics of the properties themselves) and concept-to-property features (capturing relations between each concept and each property produced). Values of each feature were averaged across concepts for each participant.

First, for each lemmatized content word produced by the participants, we used the EsPal database^117^ to derive five basic psycholinguistic features, namely: concreteness (from 1: not concrete to 7: highly concrete), imageability (from 1: not imageable to 7: highly imageable), familiarity (from 1: not familiar to 7: highly familiar), frequency (logarithmic frequency per million), and length (number of phonemes). We obtained a mean value of each of these variables per concept by averaging the values of its corresponding (lemmatized content word) properties.

Also, in line with reported procedures^23^, semantic variability was established across all content words in each property of each concept. We assigned each word to a vector in the vocabulary using FastText model, pre-trained with a large Spanish corpus. Distances between adjacent vectors were stored into a time series. Semantic consistency is computed as the variance of the text’s joint time series. When adjacent words denote distant concepts, a text has higher semantic variability.

Then, following reported procedures^23^, we used Python’s NLTK library to access WordNet, a hierarchical graph of noun-nodes leading from the top node ‘entity’ to progressively more specific concepts (e.g., ‘animal’, ‘dog’, ‘bulldog’). Each noun, detected with TreeTagger (see *Response coding and preprocessing* section), yielded a granularity score defined as the distance between its node and ‘entity’, yielding *n* distance bins (e.g., bin-3 words are closer to ‘entity’ than bin-10 words, the former indicating less precise concepts)^23^. For this analysis, Spanish responses were automatically translated into English, as in previous research^23^.

Second, we extracted concept-to-property features. To measure property distance flow, we assigned unique words to a vector using the same FastText model explained above. This vector value is assigned for both concepts and properties (in the latter case, by averaging the vectors of each property’s words). Distance between property vectors and the corresponding concept were calculated for all properties and variance was then computed for each concept. Thus, a Property distance flow value was obtained for each concept per participant.

Also, the correlational structure between each concept and each related property was calculated based on relevance and distinctiveness^118^. Relevance captures how informative each feature is for the identity of its concept (e.g. ‘*yellow*’ is more informative for the concept ‘sun’ than ‘S*unday*’). Distinctiveness is a continuous measure that parametrizes the number of concepts connected to certain property (e.g., the property ‘*it’s warm*’ could be shared by the concept ‘bed’ and ‘sun’, thus having low distinctiveness for them). Following reported metrics^118^ relevance and distinctiveness were computed via this equation:

1 $${k}_{{ij}}={x}_{{ij}}\log (I/{I}_{j})$$

where, *K*_*ij*_ represent the relevance value of a property *j* for a concept *i*, *x*_*ij*_ is the production frequency of property *j* over concept *i, I* is the total number of concepts in our dataset (*I* = 10), and *I*_*j*_ represents the number of concepts of the database for which property *j* was listed. Note that log(I/*I*_*j*_) is equivalent to distinctiveness^118^.

### Behavioral data analysis

First, each property-specific and each concept-to-property feature was compared among groups via a one-way ANCOVA with the factor ‘group’ (PD patients, bvFTD patients, HCs) and ‘years of education’ as a covariate (Fig. 1b1). To this end, we calculated each variable’s mean value per concept. For each variable, data points outside an inter-quartile range of 3 were considered outliers and removed from analyses (this resulted in the elimination of 6.9% of all data points across groups). Alpha levels were set at *p* < 0.05. Significant effects were further explored through Tukey’s HSD tests for post hoc comparisons. Effect sizes were calculated via partial eta-squared (η_p_^2^) for main effects, and Cohen’s *d* for pairwise comparisons. These analyses were performed on R (version 1.4.1717).

Second, to explore the sensitivity of our approach for probabilistic subject-level discrimination, we ran machine learning analyses to classify between (a) PD patients and HCs, (b) bvFTD patients and HCs, and (c) PD and bvFTD patients (Fig. 1b2). These analyses were performed considering all property-specific and all concept-to-property features together. In each binary classifier, data were randomly divided into five folds for stratified cross-validation, preserving the proportion of labels per group^119^ with four folds used for training and one for testing. Values for each feature were normalized using the min-max method^55^. We used a gradient boosting machine (GBM) classifier library called XGBoost (eXtreme Gradient Boosting)^54^, obtained by applying hyperparameter optimization^54^. GBM is a method that fits multiple decision trees and makes the final prediction taking the weighted sum of the predictions made by the previous trees. XGBoost implements parallel computation and regularized boosting, thus being less affected than standard algorithms by overfitting as well as correlated and irrelevant features^120^. Both GBM and XGBoost have proven sensitive to capture word-property anomalies in neurological disorders^23,121,122^. Classifier performance was reported as the mean and *SD* obtained upon 1000 iterations with different random partitions of the data. All analyses were performed on Python 3.9 and the Scikit-learn (https://scikit-learn.org/) package.

### Correlations with clinical measures

To estimate whether sensitive word properties predicted relevant neuropsychological outcomes, participants’ mean values in each feature yielding significant group effects were correlated with their scores on the MoCA and the Hayling test (Fig. 1c1). To increase variance and statistical power, these analyses were conducted collapsing each patient group with HCs (i.e., PD-HC tandem, bvFTD-HC tandem). In an exploratory fashion, we also performed correlations with the total PDQ-39 score and the PDQ-39 mobility score via Pearson’s or Spearman’s indices, as required by the distribution of data in each correlation. Correlation analyses were performed on R (version 1.4.1717).

### Neuroimaging analyses

Rs-fMRI recordings were obtained from 18 PD patients, 13 bvFTD patients, and 21 HCs matched for sex, handedness, age, and education (Supplementary material 6). Recordings were performed in two centers’ scanners, with minimally different acquisition parameters (Supplementary material 7). Recording site was entered as a covariate in all neuroimaging analysis. During the session, participants were instructed to keep not to think about anything in particular and to remain calm, awake, and with eyes closed.

Following robust methods in neurodegeneration research^121–125^, we used seed analysis to measure the functional connectivity of the bilateral sensorimotor network, the salience network, and the semantic network. Connectivity maps were averaged among the seeds of each network to derive connectivity strength values, which were captured by the weighted Symbolic Dependence Metric (wSDM), a sensitive method for neurodegenerative disorders^121,122,126^. This metric assesses the local and global temporal characteristics of the blood-oxygen-level-dependent signal by weighing a robust copula-based dependence metric by symbolic similarity. Importantly, wSDM targets dynamic nonlinear associations, a central aspect of neural connectivity that escapes traditional connectivity metrics. Indeed, wSDM outperforms Pearson’s *R* in identifying abnormalities in neurodegenerative patients^126^.

Preprocessing was performed on the Data Processing Assistant for Resting-State fMRI (DPARSF v.6.1) toolbox^127^, employing Resting-State fMRI Data Analysis Toolkit (REST v.1.8)^128^ and SPM12^129^ functions. First, to ensure magnetization stabilization, we deleted the first five volumes of each recording before starting with preprocessing steps. Second, images were slice-time corrected, referenced to the central slice of each volume, and realigned to the first scan of the recording to control the artefactual effect of head movements. Third, images were normalized to the standard MNI space utilizing the Echo-Planar Imaging template provided by SPM12 toolbox. Fourth, bandpass filtering from 0.01 to 0.1 Hz, and smoothing at 8-mm full-width-at-half-maximum isotropic Gaussian kernel were applied. Finally, to reduce the confounding effects of physiological and motion artifacts, global signals, cerebrospinal fluid, white matter, and six motion parameters were regressed. Cerebrospinal fluid and white matter masks were obtained from the tissue segmentation of the subject’s T1 recording in native space. As an additional control for head movements, mean translation and rotation were derived from the realignment step and matched between patient groups and HCs (*p* < 0.05).

For data processing, we placed two seeds per network, one in each hemisphere. These were located in main hubs of each network, on cubic regions of interest of 7 x 7 x 7 voxels^130^, based on the following MNI space coordinates: (a) primary motor cortex for the sensorimotor network (*x* = −32, *y* = −30, *z* = 68; and *x* = 32, *y* = −30, *z* = 68)^131^, (b) dorsal anterior cingulate cortex for the salience network (*x* = 10, *y* = 34, *z* = 24; and *x* = -10, *y* = 34, *z* = 24)^132^, and (c) ventral anterior temporal lobe for the semantic network (*x* = -51, *y* = 6, *z* = -39; and *x* = 51, *y* = 6, *z* = -39)^133^. Then, we utilized standard masks of each resting-state network^134^ to seclude putative brain regions considered. Finally, we averaged the connectivity values of the seeds within their respective masks (i.e., left seed with left mask, right seed with right mask) between both hemispheres, obtaining a wSDM connectivity strength score per network, per subject.

For data analysis, the connectivity strength values of each patient group were compared with those of HCs via ANCOVAs, covarying for acquisition center. Then, we examined associations between each discriminative word property with the connectivity strength of each network yielding significant between-group differences. We employed partial correlation analyses, again controlling for acquisition center (Fig. 1c2), collapsing patient groups and HCs into tandems to increase sample size, statistical power, and data variance^135–140^. Pearson’s or Spearman’s partial correlation tests were performed based on the variables’ normal or non-normal distributional form, respectively, as shown by Shapiro-Wilk test results.

### Reporting summary

Further information on research design is available in the Nature Research Reporting Summary linked to this article.

### Supplementary information

Supplementary material

Reporting summary

## Data Availability

All the data that support the findings of this study are fully available online at: https://osf.io/8pufk/.

## Code availability

All code used in this study for data preprocessing and analysis is fully available online at: https://osf.io/8pufk/.
