# Author Correction: Neurocognitive correlates of semantic memory navigation in Parkinson’s disease

**DOI:** 10.1038/s41531-024-00644-y

**Published:** 2024-01-29

**Authors:** Felipe Diego Toro-Hernández, Joaquín Migeot, Nicolás Marchant, Daniela Olivares, Franco Ferrante, Raúl González-Gómez, Cecilia González Campo, Sol Fittipaldi, Gonzalo M. Rojas-Costa, Sebastian Moguilner, Andrea Slachevsky, Pedro Chaná Cuevas, Agustín Ibáñez, Sergio Chaigneau, Adolfo M. García

**Affiliations:** 1 Graduate Program in Neuroscience and Cognition, Federal University of ABC, São Paulo, Brazil; 2 Center for Social and Cognitive Neuroscience, School of Psychology, Universidad Adolfo Ibáñez, Santiago, Chile; 3 Latin American Brain Health Institute, Universidad Adolfo Ibáñez, Santiago, Chile; 4 Laboratorio de Neuropsicología y Neurociencias Clínicas, Universidad de Chile, Santiago, Chile; 5 Cognitive Neuroscience Center, Universidad de San Andrés, Buenos Aires, Argentina; 6 National Scientific and Technical Research Council, Buenos Aires, Argentina; 7 Facultad de Ingeniería, Universidad de Buenos Aires, Buenos Aires, Argentina; 8 Global Brain Health Institute, University of California, San Francisco, California, USA; & Trinity College, Dublin, Ireland; 9 Department of Radiology, Clínica las Condes, Santiago, Chile; 10 Advanced Epilepsy Center, Clínica las Condes, Santiago, Chile; 11 Join Unit FISABIO-CIPF, Valencia, Spain; 12 School of Medicine, Finis Terrae University, Santiago, Chile; 13 Health Innovation Center, Clínica Las Condes, Santiago, Chile; 14 Memory and Neuropsychiatric Center (CMYN), Neurology Department, Hospital del Salvador & Faculty of Medicine, University of Chile, Santiago, Chile; 15 Geroscience Center for Brain Health and Metabolism (GERO), Santiago, Chile; 16 Neuropsychology and Clinical Neuroscience Laboratory (LANNEC), Physiopatology Program – Institute of Biomedical Sciences (ICBM), Neuroscience and East Neuroscience Departments, Faculty of Medicine, University of Chile, Santiago, Chile; 17 Neurology and Psychiatry Department, Clínica Alemana-Universidad Desarrollo, Santiago, Chile; 18 Facultad de Ciencias Médicas, Universidad de Santiago de Chile, Santiago, Chile; 19 Center for Cognition Research, School of Psychology, Universidad Adolfo Ibáñez, Santiago, Chile; 20 Departamento de Lingüística y Literatura, Facultad de Humanidades, Universidad de Santiago de Chile, Santiago, Chile

**Keywords:** Diagnostic markers, Parkinson's disease

Correction to: *npj Parkinson’s Disease* 10.1038/s41531-024-00630-4, published online 9 January 2024

In this article the funding from ‘Latin American Brain Health Institute (BrainLat), Universidad Adolfo Ibáñez, Santiago, Chile, #BL-SRGP2021-01’ for author Adolfo M. García was omitted. The original article has been corrected.

