# Supplementary material for: Neurocognitive correlates of semantic memory navigation in Parkinson’s disease
Source: NPJ Parkinsons Dis. 2024 Jan 9;10:15. doi: 10.1038/s41531-024-00630-4 (PMC10776628; doi:10.1038/s41531-024-00630-4)
Supplement: Supplementary file 1 — Supplementary material [file 41531_2024_630_MOESM1_ESM.pdf]

Supplementary material

1. Supplementary ANCOVA results

Supplementary Table 1. ANCOVA results.

	**PD** $N = 20$	**bvFTD** $N = 16$	**HCs** $N = 26$	**Main Effect**	**MSE**	**Pairwise comparisons (for significant main effects)** Groups	Lower	upper	*p*-value
Concreteness	4.69 (0.28)	4.51 (0.24)	4.51 (0.20)	$F = 4.27$ $p = .019^a$	0.236	PD-HCs	0.018	0.355	$.027^b$
						bvFTD-HCs	-0.181	0.179	$< .99^b$
						PD-bvFTD	-0.003	0.377	$.054^b$
Imageability	5.15 (0.31)	4.97 (0.30)	4.94 (0.27)	$F = 3.42$ $p = .039^a$	0.284	PD-HCs	0.009	0.421	$.039^b$
						bvFTD-HCs	-0.190	0.250	$.943^b$
						PD-bvFTD	-0.048	0.417	$.144^b$
Semantic variability	0.020 (0.01)	0.022 (0.01)	0.032 (0.01)	$F = 6.98$ $p < .01^a$	0.001	PD-HCs	-0.020	-0.003	$< .01^b$
						bvFTD-HCs	-0.019	-0.001	$.020^b$
						PD-bvFTD	-0.011	0.008	$.920^b$

Data presented as mean (*SD*). PD: Parkinson's disease; bvFTD: behavioral variant frontotemporal dementia; HCs: healthy controls; (a) *p*-value calculated via one-way ANCOVA; (b) *p*-value calculated via Tukey's HSD test.

2. Supplementary results from the multi-feature analysis

Supplementary Table 2. Multi-feature analysis.

**Groups**	**AUC**	**Accuracy**	**Precision**	**Recall**	**F1**	**UAR**
PD patients vs. HCs	0.77 (0.15)	0.66 (0.12)	0.65 (0.22)	0.58 (0.25)	0.60 (0.15)	0.66 (0.13)
bvFTD patients vs. HCs	0.56 (0.18)	0.61 (0.14)	0.52 (0.33)	0.34 (0.25)	0.48 (0.15)	0.56 (0.14)

Data reported as mean (*SD*). AUC: area under the ROC curve; bvFTD: behavioral variant frontotemporal dementia; HCs: healthy controls; PD: Parkinson's disease; UAR: unweighted average recall.

3. Supplementary correlation results

Supplementary Table 3. Correlations between significant word properties and neuropsychological variables.

	**PD-HC tandem**		**bvFTD-HC tandem**	
	***p*-value**	***r***	***p*-value**	***r***
**MoCA**				
Concreteness	.022[a]	- .34	.817[b]	- .04
Imageability	.046[b]	- .30	.522[b]	- .10
Semantic variability	.262[b]	.17	.911[b]	.02
**Hayling**				
Concreteness	.513[a]	.09	.926[a]	.02
Imageability	.816[a]	.04	.926[a]	.02
Semantic variability	.045[a]	- .30	.045[a]	- .32

PD: Parkinson's disease; bvFTD: behavioral variant frontotemporal dementia; HCs: healthy controls; MoCA: Montreal Cognitive Assessment. (a) *p*-value calculated via Spearman's coefficient; (b) *p*-value calculated via Pearson's coefficient.

Supplementary Table 4. Correlations between significant word properties and PDQ-39 scores.

**Correlations**	**PDQ-39 total score**		**PDQ-39 mobility score**	
	***p*-value**	***r***	***p*-value**	***r***
Concreteness	.45[a]	-.20	.88 [a]	-.04
Imageability	.30[a]	-.27	.67 [a]	-.12
Semantic variability	.72[a]	-.10	.94 [a]	-.02

(a) *p*-value calculated via Spearman's coefficient.

4. Brain-behavior correlations

Supplementary Table 5. Correlations between significant linguistic features and hypoconnected networks.

	PD-HC tandem		bvFTD-HC tandem	
	***p*-value**	***r***	***p*-value**	***r***
**Sensorimotor network**				
Concreteness	.02[a]	-.38	-	-
Imageability	.02[a]	-.39	-	-
Semantic variability	.02[a]	.38	-	-
**Salience network**				
Concreteness	< .05[a]	-.32	.05[a]	-.34
Imageability	.06[b]	-.31	.19[a]	-.23
Semantic variability	.03[b]	.35	.02[a]	.41
**Semantic network**				
Concreteness	.13[a]	-.25	-	-
Imageability	.07[b]	-.30	-	-
Semantic variability	.10[b]	-.27	-	-

PD: Parkinson's disease; bvFTD: behavioral variant frontotemporal dementia; HCs: healthy controls. (a) *p*-value calculated via Spearman's coefficient; (b) *p*-value calculated via Pearson's coefficient.

5. Power estimation

To determine the sample size required for our analyses, we ran a power estimation on G*Power software. Based on a one-way ANCOVA with three groups, we considered the following parameters: (a) alpha level of $p = .05$, (b) an effect size of $\eta^2 = .25$, and (c) power of .80. This analysis showed that a sample of 33 was adequate to reach reliable effects. The actual sample size of our study ($n = 62$) reaches a power of .98.

6. Demographic and neuropsychological data for the participants with fMRI recordings

Supplementary Table 6. Characteristics of participants with fMRI recordings.

	**PD** $N = 18$	**bvFTD** $N = 13$	**HCs** $N = 21$	**Main Effect**	**Pairwise comparisons (for significant main effects)** Groups	*p*-value
**Demographic data**						
Sex (F:M)	9:9	4:9	12:9	$\chi^2 = 2.28$ $p = 0.32^{b}$	HCs-PD<br>HCs-bvFTD<br>PD-bvFTD	-<br>-<br>-
Age	74.06 (6.76)	67.62 (12.95)	71.90 (4.54)	$F = 2.43$ $p = 0.10^{a}$	HCs-PD<br>HCs-bvFTD<br>PD-bvFTD	-<br>-<br>-
Occupation (R:A)	12:6	7:5	11:8	$\chi^2 = 0.36$ $p = 0.84^{b}$	HCs-PD<br>HCs-bvFTD<br>PD-bvFTD	-<br>-<br>-
Years since diagnosis	2.92 (2.71)	2.23 (2.34)	-	$t = 0.12$ $p = 0.90^{d}$	-	-
Years of education	10.00 (5.32)	13.31 (5.62)	12.76 (3.95)	$F = 2.22$ $p = 0.12^{a}$	HCs-PD<br>HCs-bvFTD<br>PD-bvFTD	-<br>-<br>-
**Neuropsychological data**						
MoCA	20.18 (4.76)	21.55 (4.93)	24.90 (2.30)	$F = 7.22$ $p < .01^{a}$	HCs-PD<br>HCs-bvFTD<br>PD-bvFTD	$< .01^{c}$<br>$.07^{c}$<br>$.64^{c}$
Hayling Test	14.33 (9.76)	17.91 (12.33)	8.52 (5.43)	$F = 4.49$ $p = 0.02^{a}$	HCs-PD<br>HCs-bvFTD<br>PD-bvFTD	$.12^{c}$<br>$.02^{c}$<br>$.55^{c}$

Data presented as mean (*SD*), except for sex and occupation. PD: Parkinson's disease; bvFTD: behavioral variant frontotemporal dementia; HCs: healthy controls; MoCA: Montreal Cognitive Assessment; R: retired; A: active. (a) *p*-value calculated via one-way ANOVA; (b) *p*-value calculated via chi-squared test ($\chi2$); (c) *p*-value calculated via Tukey's HSD test; (d) *p*-value calculated via two-tailed *t*-test.

7. fMRI acquisition parameters in each recruiting center

Center 1. Functional spin echo volumes were obtained in a 3T Phillips scanner with a standard head coil, parallel to the anterior-posterior commissures, covering the whole brain, were sequentially and ascendingly acquired with the following parameters: matrix dimension = 80 × 80 × 49; 49 slices; slice thickness = 3 mm; voxel size in plane = 3 mm × 3 mm × 3 mm; flip angle = 90°; repetition time = 2640 ms; echo time = 30 ms; number of volumes = 220; sequence duration = 10 min. For localization purposes, structural T1 scans were acquired, parallel to the anterior-posterior commissures, covering the whole brain, considering the following acquisition parameters: matrix dimension = 224 × 224 × 160; 160 slices; voxel size = 1 mm × 1 mm × 1 mm; flip angle = 8°; repetition time = 8300 ms; echo time = 3800 ms.

Center 2. Functional EP2D-BOLD pulse sequences were obtained in a 3T Siemens Skyra scanner with a standard head coil, parallel to the anterior-posterior commissures, covering the whole brain, were acquired sequentially intercalating pair-ascending first with the following parameters: matrix dimension = 76 × 76 × 46; 46 slices; slice thickness = 3 mm; voxel size in plane = 3 mm × 3 mm × 3 mm; flip angle = 90°; repetition time = 2660 ms; echo time = 30 ms; number of volumes = 300; sequence duration = 13.3 min. For localization purposes, structural T1 scans were acquired, parallel to the anterior-posterior commissures, covering the whole brain, considering the following acquisition parameters: matrix dimension = 224 × 224 × 208; 208 slices; voxel size = 1 mm × 1 mm × 1 mm; flip angle = 8°; repetition time = 1700 ms; echo time = 2000 ms.
