# Supplementary material for: Neurocognitive correlates of semantic memory navigation in Parkinson’s disease
Source: NPJ Parkinsons Dis. 2024 Jan 9;10:15. doi: 10.1038/s41531-024-00630-4 (PMC10776628; doi:10.1038/s41531-024-00630-4)
Supplement: Supplementary file 2 — Reporting summary [file 41531_2024_630_MOESM2_ESM.pdf]

nature portfolio

Corresponding author(s): Adolfo M. García.
DAPR | NPJPARKD-02070R1

Last updated by author(s): Dec 27, 2023

Reporting Summary

Nature Portfolio wishes to improve the reproducibility of the work that we publish. This form provides structure for consistency and transparency in reporting. For further information on Nature Portfolio policies, see our Editorial Policies and the Editorial Policy Checklist.

Statistics

For all statistical analyses, confirm that the following items are present in the figure legend, table legend, main text, or Methods section.

n/a	Confirmed	
☐	☒	The exact sample size (*n*) for each experimental group/condition, given as a discrete number and unit of measurement
☐	☒	A statement on whether measurements were taken from distinct samples or whether the same sample was measured repeatedly
☐	☒	The statistical test(s) used AND whether they are one- or two-sided<br>*Only common tests should be described solely by name; describe more complex techniques in the Methods section.*
☐	☒	A description of all covariates tested
☐	☒	A description of any assumptions or corrections, such as tests of normality and adjustment for multiple comparisons
☐	☒	A full description of the statistical parameters including central tendency (e.g. means) or other basic estimates (e.g. regression coefficient) AND variation (e.g. standard deviation) or associated estimates of uncertainty (e.g. confidence intervals)
☐	☒	For null hypothesis testing, the test statistic (e.g. *F*, *t*, *r*) with confidence intervals, effect sizes, degrees of freedom and *P* value noted<br>*Give P values as exact values whenever suitable.*
☒	☐	For Bayesian analysis, information on the choice of priors and Markov chain Monte Carlo settings
☒	☐	For hierarchical and complex designs, identification of the appropriate level for tests and full reporting of outcomes
☐	☒	Estimates of effect sizes (e.g. Cohen's *d*, Pearson's *r*), indicating how they were calculated

Our web collection on statistics for biologists contains articles on many of the points above.

Software and code

Policy information about availability of computer code

Data collection	Audio recordings were obtained via WaveSurfer 1.8.8p4.
Data analysis	Statistical analyses were performed on R, an open-source statistical package.

For manuscripts utilizing custom algorithms or software that are central to the research but not yet described in published literature, software must be made available to editors and reviewers. We strongly encourage code deposition in a community repository (e.g. GitHub). See the Nature Portfolio guidelines for submitting code & software for further information.

Data

Policy information about availability of data

All manuscripts must include a data availability statement. This statement should provide the following information, where applicable:
- Accession codes, unique identifiers, or web links for publicly available datasets
- A description of any restrictions on data availability
- For clinical datasets or third party data, please ensure that the statement adheres to our policy

The datasets generated and/or analyzed during the current study are available in the Open Science Framework (OSF) repository under the title “Semantic memory navigation in early stage Parkinson’s disease: Word-property markers and neurocognitive signatures”, https://osf.io/8pufk/

Research involving human participants, their data, or biological material

Policy information about studies with human participants or human data. See also policy information about sex, gender (identity/presentation), and sexual orientation and race, ethnicity and racism.

Reporting on sex and gender	The term 'sex' has been used to indicate a biological attribute. Findings apply to both sexes, as noted in the manuscript. Sex was determined via self-reports, as noted in the manuscript. Disaggregated sex data has been provided in the source data. Our sample includes 10 women and 10 men with Parkinson's disease, 5 women and 11 men with behavioral variant frontotemporal dementia, as well as 16 women and 10 men in healthy condition (as reported in Table 1). No separate analyses were performed on each sex given that no specific hypotheses were raised for this variable in the study design.
Reporting on race, ethnicity, or other socially relevant groupings	Sociodemographic variables were matched between patients and controls. Because years of education differed between groups, this variable was entered as a covariate by ANCOVA in all behavioral analyses. This variable was not considered confounding in the neuroimaging analyses, as it did not differ between groups in this subsample (as reported in Table S5).
Population characteristics	As detailed in Table 1, Parkinson's disease patients had a mean of 74.25 years of age, 9.95 years of education, 2 years since diagnosis, 72.19 PDQ-39 score, 14.90 Hayling test score, and 20.05 MoCA score. Behavioral variant frontotemporal dementia patients had a mean of 68.50 years of age, 14 years of education, 2.79 years since diagnosis, 15.79 Hayling test score, and 22.07 MoCA score. Healthy controls had a mean of 71.73 years of age, 13 years of education, 8 Hayling test score, and 24.33 MoCA score.
Recruitment	Participants were recruited from an ongoing protocol at the Memory and Neuropsychiatry Clinic in Santiago, Chile, by postings and talks organized to invite participants.
Ethics oversight	The study was approved by the Institutional Ethics Committee.

Note that full information on the approval of the study protocol must also be provided in the manuscript.

Field-specific reporting

Please select the one below that is the best fit for your research. If you are not sure, read the appropriate sections before making your selection.

☐ Life sciences ☒ Behavioural & social sciences ☐ Ecological, evolutionary & environmental sciences

For a reference copy of the document with all sections, see nature.com/documents/nr-reporting-summary-flat.pdf

Behavioural & social sciences study design

All studies must disclose on these points even when the disclosure is negative.

Study description	The study is quantitative experimental.
Research sample	Parkinson's disease, behavioral variant frontotemporal dementia patients, and healthy controls. Parkinson's disease patients had a mean of 74.25 years of age, 9.95 years of education, 2 years since diagnosis, 72.19 PDQ-39 score, 14.90 Hayling test score, and 20.05 MoCA score. Behavioral variant frontotemporal dementia patients had a mean of 68.50 years of age, 14 years of education, 2.79 years since diagnosis, 15.79 Hayling test score, and 22.07 MoCA score. Healthy controls had a mean of 71.73 years of age, 13 years of education, 8 Hayling test score, and 24.33 MoCA score. The study was chosen given its compliance with inclusion/exclusion criteria and accessibility for testing.
Sampling strategy	A convenience sampling strategy was employed. The sample size surpasses the mean of previous linguistic studies on Parkinson's disease, and it allowed for cross-validation analyses, guaranteeing generalizability of results. Detection of robust results in previous studies with similar or smaller sample sizes pointed to the adequacy of the present sample size.
Data collection	A microphone and audio software were used for speech data acquisition. Neuropsychological and demographic data were obtained with pen and paper tests. Nobody was present during testing other than the participants and the examiner. The examiner was blind to the study hypotheses during data collection.
Timing	Start: 09/08/2017 | Finish: 12/02/2019
Data exclusions	No data were excluded from the analyses.
Non-participation	No participants dropped out or declined participation
Randomization	Participants were allocated into the Parkinson's disease, behavioral variant frontotemporal dementia, or healthy control groups based on their health status.

Reporting for specific materials, systems and methods

We require information from authors about some types of materials, experimental systems and methods used in many studies. Here, indicate whether each material, system or method listed is relevant to your study. If you are not sure if a list item applies to your research, read the appropriate section before selecting a response.

Materials & experimental systems

n/a	Involved in the study
☒	☐ Antibodies
☒	☐ Eukaryotic cell lines
☒	☐ Palaeontology and archaeology
☒	☐ Animals and other organisms
☐	☒ Clinical data
☒	☐ Dual use research of concern
☒	☐ Plants

Methods

n/a	Involved in the study
☒	☐ ChIP-seq
☒	☐ Flow cytometry
☐	☒ MRI-based neuroimaging

Clinical data

Policy information about clinical studies

All manuscripts should comply with the ICMJE guidelines for publication of clinical research and a completed CONSORT checklist must be included with all submissions.

Clinical trial registration	Not applicable.
Study protocol	Not applicable.
Data collection	Not applicable.
Outcomes	Not applicable.

Magnetic resonance imaging

Experimental design

Design type	Resting state.
Design specifications	A single block of about 10 minutes.
Behavioral performance measures	Not applicable.

Acquisition

Imaging type(s)	Functional and structural.
Field strength	3 Tesla.
Sequence & imaging parameters	As reported in supplementary material 6, for center 1, functional spin echo volumes were obtained in a 3T Phillips scanner with a standard head coil, parallel to the anterior-posterior commissures, covering the whole brain, were sequentially and ascendingly acquired with the following parameters: matrix dimension = 80 × 80 × 49; 49 slices; slice thickness = 3 mm; voxel size in plane = 3 mm × 3 mm × 3 mm; flip angle = 90°; repetition time = 2640 ms; echo time = 30 ms; number of volumes = 220; sequence duration = 10 min. For localization purposes, structural T1 scans were acquired, parallel to the anterior-posterior commissures, covering the whole brain, considering the following acquisition parameters: matrix dimension = 224 × 224 × 160; 160 slices; voxel size = 1 mm × 1 mm × 1 mm; flip angle = 8°; repetition time = 8300 ms; echo time = 3800 ms. Regarding center 2, functional EP2D-BOLD pulse sequences were obtained in a 3T Siemens Skyra scanner with a standard head coil, parallel to the anterior-posterior commissures, covering the whole brain, were acquired sequentially intercalating pair-ascending first with the following parameters: matrix dimension = 76 × 76 × 46; 46 slices; slice thickness = 3 mm; voxel size in plane = 3 mm × 3 mm × 3 mm; flip angle = 90°; repetition time = 2660 ms; echo time = 30 ms; number of volumes = 300; sequence duration = 13.3 min. For localization purposes, structural T1 scans were acquired, parallel to the anterior-posterior commissures, covering the whole brain, considering the following acquisition parameters: matrix dimension = 224 × 224 × 208; 208 slices; voxel size = 1 mm × 1 mm × 1 mm; flip angle = 8°; repetition time = 1700 ms; echo time = 2000 ms.
Area of acquisition	Whole brain.

Diffusion MRI ☐ Used ☒ Not used

Preprocessing

Preprocessing software	Preprocessing was performed on the Data Processing Assistant for Resting-State fMRI (DPARSF v.6.1) toolbox, employing Resting-State fMRI Data Analysis Toolkit (REST v.1.8) and SPM12 functions.
Normalization	Images were normalized to the standard MNI space utilizing the Echo-Planar Imaging template provided by SPM12 toolbox.
Normalization template	Images were normalized to the standard MNI space.
Noise and artifact removal	To reduce the confounding effects of physiological and motion artifacts, global signals, cerebrospinal fluid, white matter, and six motion parameters were regressed. Cerebrospinal fluid and white matter masks were obtained from the tissue segmentation of the subject's T1 recording in native space. As an additional control for head movements, mean translation and rotation were derived from the realignment step and matched between patient groups and HCs ($p < 0.05$).
Volume censoring	To control for head movements, six motion parameters were regressed and mean translation and rotation were matched between patient groups and HCs ($p < 0.05$).

Statistical modeling & inference

Model type and settings	Univariate (inferential) models.
Effect(s) tested	For data analysis, the connectivity strength values of each patient group were compared with those of HCs via ANCOVAs, covarying for acquisition center. Then, we examined associations between each discriminative word property with the connectivity strength of each network yielding significant between-group differences. We employed partial correlation analyses, again controlling for acquisition center, collapsing patient groups and HCs into tandems to increase sample size, statistical power, and data variance. Pearson's or Spearman's partial correlation tests were performed based on the variables' normal or non-normal distributional form, respectively, as shown by Shapiro-Wilk test results.

Specify type of analysis: ☐ Whole brain ☒ ROI-based ☐ Both

Anatomical location(s)	We placed two seeds per network, one in each hemisphere. These were located in main hubs of each network, on cubic regions of interest of 7x7x7 voxels1, based on the following MNI space coordinates: (a) primary motor cortex for the sensorimotor network (x = −32, y = −30, z = 68; and x = 32, y = −30, z = 68), (b) dorsal anterior cingulate cortex for the salience network (x = 10, y = 34, z = 24; and x = -10, y = 34, z = 24), and (c) ventral anterior temporal lobe for the semantic network (x = -51, y = 6, z = -39; and x = 51, y = 6, z = -39).
Statistic type for inference (See Eklund et al. 2016)	We utilized standard masks of each resting-state network to seclude putative brain regions considered.
Correction	Correction for multiple comparisons was not applied.

Models & analysis

n/a	Involved in the study
☐	☒ Functional and/or effective connectivity
☒	☐ Graph analysis
☒	☐ Multivariate modeling or predictive analysis

Functional and/or effective connectivity	Connectivity maps were averaged among the seeds of each network to derive connectivity strength values, which were captured by the weighted Symbolic Dependence Metric (wSDM), a sensitive method for neurodegenerative disorders. This metric assesses the local and global temporal characteristics of the blood-oxygen-level-dependent signal by weighing a robust copula-based dependence metric by symbolic similarity. wSDM targets dynamic nonlinear associations, a central aspect of neural connectivity that escapes traditional connectivity metrics.
